# Multiple Myeloma Relapse as Intracranial Plasmacytoma: A Rare Presentation

**DOI:** 10.7759/cureus.8357

**Published:** 2020-05-30

**Authors:** Amulya Prakash, Sindhuja Korem, Sindhura Inkollu, Patrick Lee

**Affiliations:** 1 Internal Medicine, Monmouth Medical Center, Long Branch, USA; 2 Oncology, Monmouth Medical Center, Long Branch, USA

**Keywords:** multiple myeloma, relapse, plasmacytoma, intracranial

## Abstract

Multiple myeloma (MM) infrequently involves the central nervous system (CNS). The usual sites of involvement are skull and meninges; however, intracranial tumors are exceedingly rare. We report the case of a 60-year-old female who presented to our center for the complaint of recurrent syncope. The patient was diagnosed with MM approximately one and a half years ago and had received chemotherapy followed by an allogeneic bone marrow transplant and was in remission afterward. We initiated workup for syncope and a brain MRI revealed an intracranial mass. Histopathological studies of the intracranial mass confirm the diagnosis of plasmacytoma and further testing shows relapse of MM. This is a unique case of MM relapse with isolated intracranial plasmacytoma. It usually carries a poor prognosis. Early diagnosis and management are imperative to improve survival.

## Introduction

Multiple myeloma (MM) is a common hematological malignancy involving plasma cells, leading to their malignant proliferation and production of monoclonal paraprotein. Central nervous system (CNS) involvement is very rare in MM with an incidence of 0.7% [1]. This unique and infrequent presentation makes the diagnosis of this condition challenging. We present a rare case of MM that was believed to be in remission; which relapsed as intracranial plasmacytoma. 

## Case presentation

**Figure 1:**
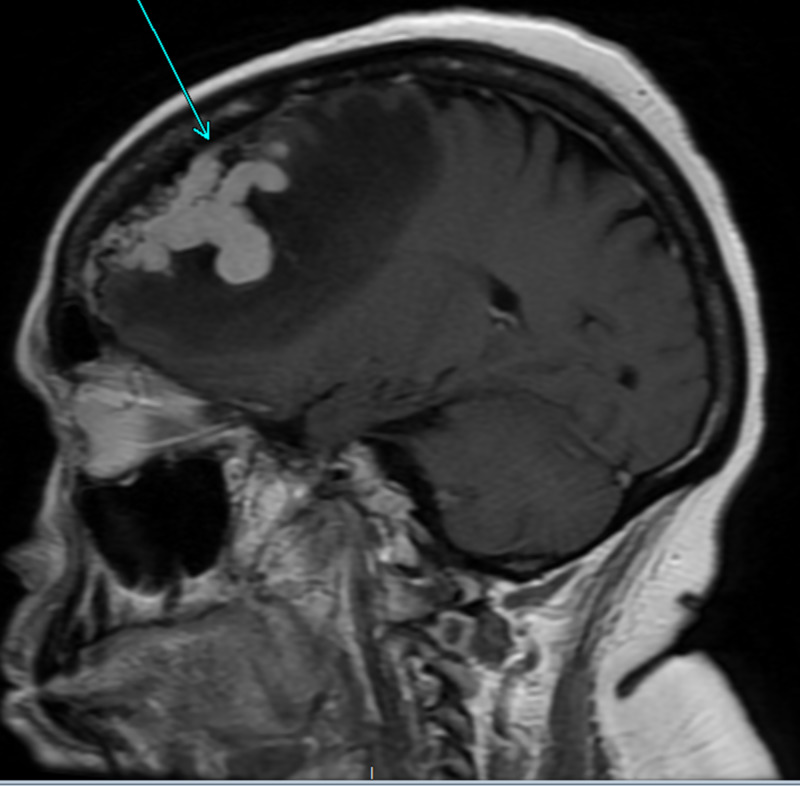
MRI brain with contrast sagittal view T1 sequence of left frontal lobe growth with surrounding vasogenic edema.

**Figure 2:**
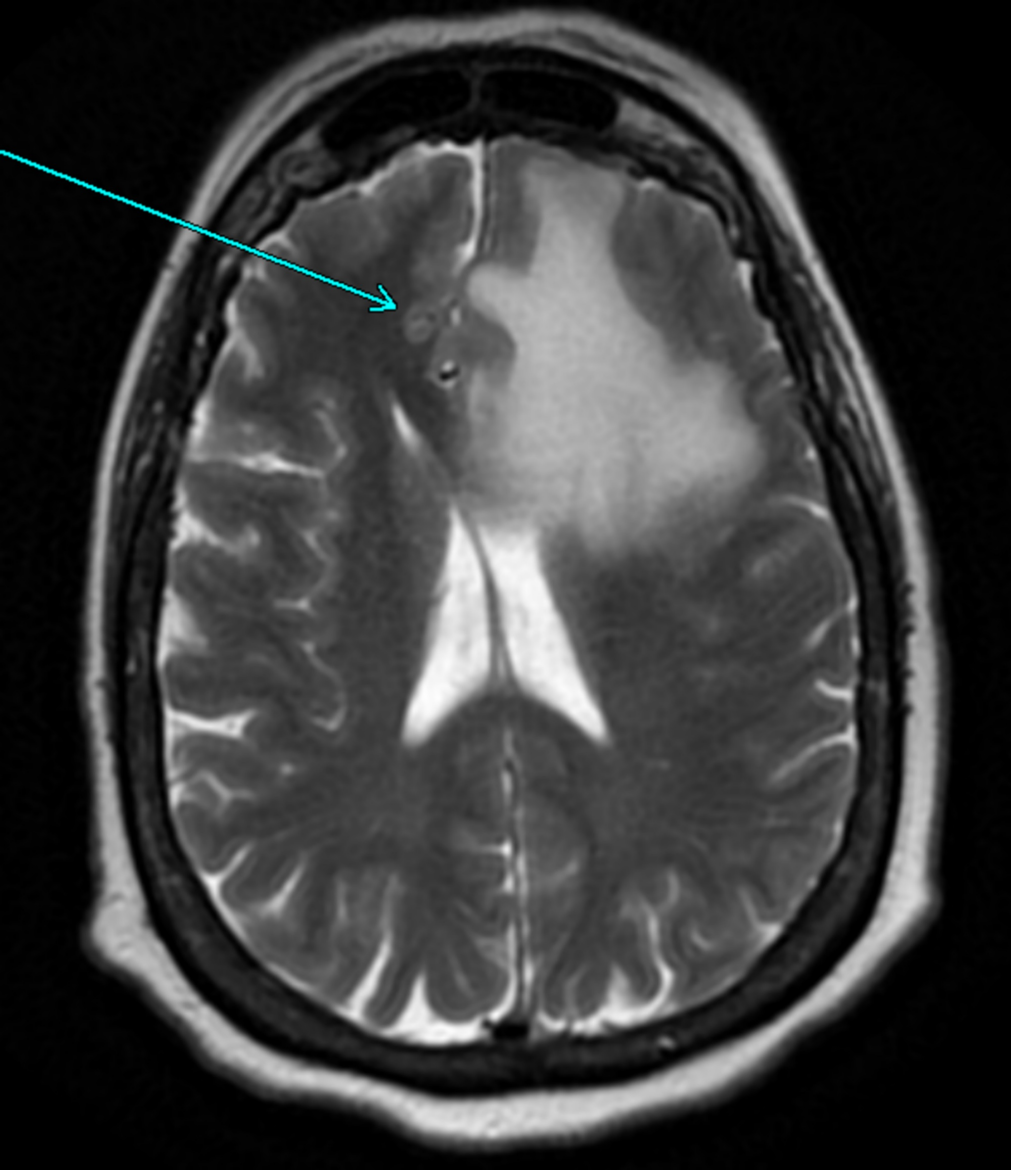
MRI brain with contrast axial view T2 sequence shows left frontal lobe growth with midline shift to right.

A 60-year-old female presented with a complaint of recurrent episodes of syncope. The patient had been diagnosed with MM 16 months ago prior to the presentation. MM was diagnosed when she presented with pathological fracture of the right distal femur and left tibial comminuted fracture. She was initially treated with bortezomib, melphalan, and dexamethasone. She received radiation therapy to right femur, right tibia, and sacrum. Six months after the initial diagnosis, she had an autologous bone marrow transplant. The patient could not be placed on any suppressive therapy due to chronic thrombocytopenia. The patient presented to our center and mentioned that she had three episodes of syncope in the last two months. She denied having any associated symptoms like lightheadedness, nausea, abnormal movements, focal weakness, paraesthesia, fecal or urinary incontinence. The patient also mentioned that she never experienced any kind of neurological symptom in the past. At the time of admission, her vitals were normal and physical examination was unremarkable. Blood tests including complete blood count and chemical metabolic panel were normal except for thrombocytopenia. MRI of the brain with contrast showed left frontal lobe anterior mass with adjacent vasogenic edema (Figure 1) and midline shift to the right as shown in Figure 2. 

Cerebrospinal fluid (CSF) analysis was negative for malignant cells. The patient underwent stereotactic left frontal craniotomy and excision of the brain tumor. Frozen sections of the excised mass were consistent with plasmacytoma. Flow cytometry demonstrates markers positive CD 138, 56, 38. Bone marrow biopsy was done later which confirmed the effacement of marrow cells by plasma cell neoplasm confirming relapse of MM. After diagnosis, the patient was started on the etoposide-prednisolone-vincristine-cyclophosphamide-hydroxydaunorubicin regimen. One-year follow up on the patient in the oncology clinic shows evidence of active disease but she denies having any further episodes of syncope.

## Discussion

Through this case, we discuss a rare complication of MM as a relapse of the disease in the form of extraosseous plasmacytoma. Extramedullary plasmacytoma is a soft-tissue plasma cell tumor and has an occurrence rate of 5% of all plasma cell neoplasms, and most often occurring in the upper respiratory tract or oral cavity [2-4]. Intracranial plasmacytomas are exceedingly rare, usually seen in cases of the relapsed disease with most cases involving the meninges (meningeal myelomatosis) or the skull rather than the brain parenchyma itself [5-6]. As per a retrospective review of 172 CNS myeloma patients done by Jurczyszyn et al., the median time from MM diagnosis to myeloma thus involvement was three years and is usually associated with disease relapse [7]. 

As per a prior study, there is an association between CNS MM relapse and autologous hematopoietic stem cell transplant where most cases were observed to be leptomeningeal disease [8]. Several cases of solitary intracranial plasmacytoma have been reported, but these are typically intraosseous tumors [5-6]. To summarize, the relapsed disease process would involve osseous and extramedullary sites more frequently and rarely intracranial.

Diagnosis is complex and requires tumor detection and characterization. Contrast-enhanced MRI brain is more sensitive than CT of the head and constitutes the method of choice in the detection of CNS MM; however, it was also associated with a false-negative rate of 10% [9]. Therefore, it is preferable to perform imaging, pathological, and CSF examination concurrently. In the case of our patient, MRI findings such as homogenous enhancement with perilesional edema were present which can also be seen in lymphoma or meningioma. Prior studies on CNS and extramedullary myeloma have identified alterations of chromosome 13 and 17, and translocation (4; 14) as higher risk features [10]. Cytological studies of her plasmacytoma displayed CD138-positive marker as seen in most CNS myeloma cases. We also noted a high percentage of proliferation marker Ki-67 which is suggestive of aggressive growth pattern and has been associated with an increased risk of extraosseous relapse of myeloma [11].

From our extensive review of literature, we could not define a standard guideline for treatment and usually, a multimodal approach is attempted. Systemic treatment, alone or combined with radiotherapy, resulted in a significant improvement of survival in patients when compared to no systemic therapy [12]. Intrathecal agents have been used in CNS MM with conflicting results as intrathecal agents are often used in combination with systemic therapies, and to this date, did not prove to be efficient as monotherapy [13]. Whole brain radiation is another therapeutic option in CNS MM but its practical application is limited due to associated toxicity.

## Conclusions

Although rare, MM can potentially involve CNS. Patients with neurological symptoms that cannot be explained otherwise should aggressively be evaluated for CNS MM with imaging, CSF cytology, flow cytometry, and histopathological studies. Patients with unfavorable cytogenetic profile, refractory course of treatment, and history of stem cell transplant are at a relatively higher risk of relapse with myelomatous involvement. Even with early diagnosis, prognosis remains dismal with an average survival of three to six months after myelomatous involvement. However, aggressive multi-modal treatment may prolong life by months.

## References

[1] Paludo J, Painuly U, Kumar S (2016). Myelomatous Involvement of the central nervous system. Clin Lymphoma Myeloma Leuk.

[2] Alexiou C, Kau RJ, Dietzfelbinger H, Kremer M, Spiess JC, Schratzenstaller B, Arnold W (1999). Extramedullary plasmacytoma: tumor occurrence and therapeutic concepts. Cancer.

[3] Dimopoulos MA, Kiamouris C, Moulopoulos LA (1999). Solitary plasmacytoma of bone and extramedullary plasmacytoma. Hematol/Oncol Clin N Am.

[4] Galieni P, Cavo M, Pulsoni A (2000). Clinical outcome of extramedullary plasmacytoma. Haematologica.

[5] Alegre A, Tomas JF, Fernández‐Ranada JM, Pinilla I, Gil‐Fernández JJ, Fernández‐Rañada JM (1996). Multiple myeloma presenting as plasmacytoma of the base of the skull. Am J Hematol.

[6] Savas MC, Benekli M, Haznedaroglu IC, Dundar SV (1997). Bulky plasmacytoma of the skull with intracranial involvement. Am J Hematol.

[7] Jurczyszyn A, Grzasko N, Gozzetti A (2016). Central nervous system involvement by multiple myeloma: a multi-institutional retrospective study of 172 patients in daily clinical practice. Am J Hematol.

[8] Petersen SL, Wagner A, Gimsing P (1999). Cerebral and meningeal multiple myeloma after autologous stem cell transplantation. A case report and review of the literature. Am J Hematol.

[9] Provenzale JM, Schaefer P, Traweek ST, Ferry J, Moore JO, Friedman AH, McLendon RE (1997). Craniocerebral plasmacytoma: MR features. Am J Neuroradiol.

[10] Fassas AB, Muwalla F, Berryman T (2002). Myeloma of the central nervous system: association with high-risk chromosomal abnormalities, plasmablastic morphology and extramedullary manifestations. Br J Haematol.

[11] Rasche L, Bernard C, Topp MS (2012). Features of extramedullary myeloma relapse: high proliferation, minimal marrow involvement, adverse cytogenetics: a retrospective single-center study of 24 cases. Ann Hematol.

[12] Nahi H, Svedmyr E, Lerner R (2020). Bendamustine in combination with high-dose radiotherapy and thalidomide is effective in treatment of multiple myeloma with central nervous system involvement. Eur J Haematol.

[13] Chamberlain MC, Glantz M (2008). Myelomatous meningitis. Cancer.

